# The challenge of COVID-19 case identification and ascertainment in sub-Saharan Africa: the case of Cameroon

**DOI:** 10.11604/pamj.supp.2020.35.24368

**Published:** 2020-06-20

**Authors:** Sylvain Raoul Simeni Njonnou, Fernando Kemta Lekpa, Christian Ngongang Ouankou, Eric Vounsia Balti, Simeon Pierre Choukem, For The United#COVID-19

**Affiliations:** 1 Department of Internal Medicine and Specialties, Faculty of Medicine and Pharmaceutical Sciences, University of Dschang, Dschang, Cameroon; 2 The University of Dschang Taskforce for the Elimination of COVID-19 (UNITED#COVID-19); 3 Department of Internal Medicine, Douala General Hospital, Douala, Cameroon; 4 Department of Internal Medicine and Specialties, Yaoundé University Teaching Hospital, Yaoundé, Cameroon; 5 Diabetes Research Center and Department of Internal Medicine, Universiteit Ziekenhuis Brussel, Vrije Universiteit Brussel, Brussels, Belgium; 6 Endocrine and Diabetes Unit and National Obesity Centre, Department of Internal Medicine, Yaoundé Central Hospital, Yaoundé, Cameroon; 7 Health and Human Development (2HD) Research Network, Douala, Cameroon

**Keywords:** COVID-19 case identification, COVID-19 ascertainment, challenge, Cameroon

## Abstract

Confirmation of COVID-19 infection is still challenging in Cameroon due to lack of sufficient RT-PCR tests and screening capacity. International organizations as well as philanthropic donators initially provided these tests. Due to limited kits availability, mass screening is currently a luxury that the country cannot afford. This impacts on estimates of disease prevalence, on the understanding of its burden and delays the implementation of targeted preventive measures. Besides RT-PCR, chest CT-scan could be a useful tool for screening purposes. However, its sensitivity and cost make its integration in management algorithms difficult. We discuss below the challenges and potential solutions that could ease the diagnosis of COVID-19 infection in Cameroon.

## Commentary

The outbreak of the new coronavirus disease (COVID-19), caused by the Severe Acute Respiratory Syndrome Coronavirus-2 (SARS-CoV-2), was declared a pandemic by the World Health Organization on March 11, 2020 [1]. Africa is among the less affected continents looking at the current total number of cases and fatalities. However, this information must be taken with a pinch of salt given the low number of tests that are carried out in Africa and particularly in Cameroon (1500 tests for a million inhabitants) [2]. This number is far from being among the largest achieved in Africa and the world. In addition, these tests are solely based on reverse transcription-polymerase chain reaction (RT-PCR). The associated sensitivity is 72% on sputum and 63% on nasal swab samples when performed in optimal conditions, creating a fairly high risk of false negatives [3]. Furthermore, these tests are currently performed only in ten laboratories in Cameroon (for a total population of 26 million inhabitants). This low number of tests could be explained by the lack of local manufacture and thus, the dependence on private donators (Jack Ma´s Foundation, Clinton Health Access Initiative and China embassy) for a part of supplies. At first, all countries worldwide faced tests shortage. This furthered the development and use of alternative diagnosis strategies which helped resolve the situation of test availability in almost all Western countries. However, limited access to testing is persisting in Sub-Saharan Africa at a moment the epidemic is on a steep rise. We found it relevant to discuss the issues related to the difficulties in COVID-19 case identification and ascertainment in Cameroon and to suggest potential solutions, all these being crucial to the effectiveness of the epidemic control strategy in the country.

**Concurrent use of chest CT-scan and SARS-CoV-2 PCR for case identification:** a mean to improve the identification of case could be the use of chest CT-scan, for selection of individuals with high risk of COVID-19. Chest CT-scan has been shown to have high sensitivity (about 97%) among COVID-19 patients with positive [4,5] and of 75% in those with negative RT-PCR, respectively [4]. The biggest pool of information about chest CT-scan sensitivity for diagnosing COVID-19 was provided by Ai et al. They retrospectively reviewed a total of 1014 patients suspected of COVID-19 and who underwent both chest CT-scan and RT-PCR (on throat swab samples). The authors calculated the sensitivity, specificity, positive predictive value (PPV), negative predictive value (NPV) and accuracy of chest CT imaging using RT-PCR results as reference. Of the 601 patients with positive RT-PCR results, 97% had positive chest CT-scan. Among the 413 patients with negative results, 75% had chest CT-scans suggestive of COVID-19. The specificity and accuracy of chest tomography in indicating the infection were respectively 25% and 68%. However, 21 patients had positive RT-PCR without lesions at the initial imaging assessment. This discrepancy could be explained by the disease phase which might had been early for those patients when chest CT-scan was performed. The use of an RT-PCR assay with a relatively low-positive rate as reference (that could overestimate the sensitivity of the CT-scan) and the type of sample (throat swab in this study, which has a low sensitivity) are obvious limitations of the study [4]. Nonetheless, these data are very attractive and support how convenient CT-scan could be for preselecting high-risk individuals for further testing with RT-PCR in settings or contexts where access to tests and related equipment is limited.

Despite these interesting results on performance of CT-scan for diagnosis of COVID-19, this solution is not the most readily available in our country. The most important challenge is the limited number of CT-scan facilities and the associated financial burden. Indeed, the cost of chest CT-scan ranges locally between 108 and 133 USD while the minimum wage in Cameroon is 60.66 USD [6]. Ultimately, it is obvious that only few people would afford not the chest CT-scan. To date, the results of SARS-CoV-2 RT-PCR in Cameroon are obtained within two to seven days. This certainly impacts the daily estimate of disease and death incidence, with consequence on decision making and ultimately on disease progression in this critical period where lockdown measures are being relaxed. Due to its high sensitivity, chest CT-scan was the diagnostic tool that was used at the beginning of the pandemic in the absence of RT-PCR. The resulting expenditure has not yet been fully assessed. However, the above-mentioned cost led to its exclusion from diagnostic schemes at least in Cameroon. This exclusion of the classic diagnostic scheme should not avoid its use in patients with severe or critical forms of COVID-19.

**Repurposing currently existing equipment dedicated to other infectious diseases:** another potential solution would consist in repurposing tools used for the monitoring of other infectious diseases such as the loop-mediated isothermal amplification (LAMP). This technology is currently used for the diagnosis and monitoring of tuberculosis in Cameroon. LAMP is a method of nucleic acid amplification that exhibits high sensitivity and specificity. It is very rapid, and does not require expensive reagents or materials, and thus is cost-effective. From this, the technology could be applied within the field at short-notice and used by staffers with even limited training. In comparison to conventional RT-PCR assays, it has shown a ten-fold higher sensitivity. In addition, the results could be provided within one hour, thus achieving significant time savings [7]. This solution, given the few number of these equipment, would however be insufficient to guarantee massive accessibility to diagnostic tests. Furthermore, caution needs to be advised regarding the potential impact of this measure to the follow-up and monitoring of these other endemic diseases which will require a tandem response with COVID-19. According to Manyazewal et al., there is an exacerbation of global Tuberculosis (TB) by the COVID-19 pandemic, as regular treatment facilities are being closed and TB could be misdiagnosed in settings where COVID-19 testing is not available [8]. However, on the one hand, countries that were the most affected by the COVID-19 health crisis so far, including the United States of America, Italy, Spain, France and Great Britain are very different from sub-Saharan African countries and thus, there is limited information on how co-infection with other infectious diseases would affect patients with COVID-19. On the other hand, these countries have less infectious diseases than countries in sub-Saharan Africa. It is therefore essential to monitor treatment programs for target infectious diseases, assess the impact of COVID-19 infection on these and maintain the effectiveness of immunizations campaigns during this period.

**Prescreening using risk scores:** what could, in our view, be more useful overall in a resource-limited country like ours, would be the development of a diagnostic probability score using clinical and biological data. A score could be derived from epidemiological and clinical findings such as age, fever, cough, dyspnea or oxygen need. This could be complemented by biological parameters including C-reactive protein, procalcitonin, D-dimers, lactate dehydrogenase, lymphocytes count, serum creatinine and troponin amongst others. The rationale for using these variables is linked to their impact on patient diagnosis or prognosis based on previous observations. For instance, age is suggested because current reports clearly indicate that elderly people are more susceptible to COVID-19 and they more frequently develop severe disease. The other parameters are chosen due to their usual association with COVID-19 infection. Clinical signs relate to features of pneumonia while biological parameters are those which were identified as the most frequently encountered in patients infected with this disease. Indeed, a weighting of these different variables will have to be carried out. Such a test would be a very cost-effective tool for screening COVID-19 infection. Moreover, concurrent use of the score with determinants of requirement of more advanced supportive therapy in intensive care unit could help identify threshold levels for disease stratification according to severity. This prediction model can be helpful to mitigate the burden on the healthcare system, while also providing the most cost-effective care for patients using an efficient diagnosis scheme. A weakness of the use of scores of disease prediction could be, in our setting, the access to biological exams due to the limited capacities of the laboratories of peripheral hospitals. The limited radiological platforms that are essential to the diagnosis but also for patient´s follow up is, therefore, a major limitation.

**Implementation of serological tests:** an alternative mean for case identification and ascertainment is the use of serological tests. These tests are, comparatively to nucleic acid detection, easier to perform and require less technical expertise and equipment. Blood samples are collected in tubes, which pose less potential risk to the staff handling the samples. Moreover, they can be performed in a basic clinical laboratory and smaller community settings, therefore reaching a wider application. One would however need to consider the onset of antibody positivity one to three weeks after the occurrence of symptoms [9]. Another pitfall is the lack of specificity of some of these tests, which increases the risk of false-negative detection. This is why, as recommended by the Center for Disease Control and Prevention (CDC), it is important to choose an assay with high specificity and testing populations as well as individuals with an elevated likelihood of previous exposure to SARS-CoV-2. Alternatively, an orthogonal testing algorithm can be used when the expected positive predictive value of a single test is low [10]. That notwithstanding, their use could be helpful not only in the case of suspected false-negative RT-PCR tests but also for the implementation of selective quarantine measures after lifting lockdown measures. Other relevant applications are: the assessment of the real penetration of the disease in populations, even once the epidemic will be controlled; and the evaluation of immunity status. To understand the relevance of the implementation of serological tests for COVID-19, one will however need to address some concerns. Among these, that of whether the production of antibodies would be the same in all individuals and whether their presence would induce immunity. Secondly, whether this immunity is partial or total and its duration will need to be questioned. And down to earth, where to purchase these tests whose quality standards must be as strict as possible. Local manufacture of these tests following strict procedures appears to be a better solution.

## Conclusion

Diagnosis of COVID-19 is still challenging in sub-Saharan Africa, particularly in Cameroon due to the lack of sufficient RT-PCR tests. The use of a screening strategy based on the conduct of low dose CT-Scan faces the same difficulty related to the cost in a setting where resources have to be managed with caution. Other alternative solutions, to optimize the identification of individuals exposed to the SARS-CoV-2 virus might be the repurposing of the tools used for monitoring other infectious diseases such as the LAMP, the development of a diagnosis model to derive a predictive score based on clinical and biological data, and the use of serological tests.

## Competing interests

The authors declare no competing interests.
